# Supplementary material for: Identification and characterization of probiotics isolated from indigenous chicken (Gallus domesticus) of Nepal
Source: PLoS One. 2023 Jan 19;18(1):e0280412. doi: 10.1371/journal.pone.0280412 (PMC9851537; doi:10.1371/journal.pone.0280412)
Supplement: S1 Table — (DOCX) [file pone.0280412.s001.docx]

**S1 Table.** Demographic details of chicken cloacal samples shown by identification number, sampling locations and the chicken breed.

| **Sample ID** | **Sampling location** | **Chicken breed** |
| --- | --- | --- |
| FARP-01 | Lakuribhanjyang-sisnekharka | Local |
| FARP-02 | Lakuribhanjyang-sisnekharka | Giriraj |
| FARP-03 | Lakuribhanjyang-sisnekharka | Local |
| FARP-04 | Lakuribhanjyang-sisnekharka | Local |
| FARP-05 | Lakuribhanjyang-sisnekharka | Local |
| FARP-06 | Lakuribhanjyang-sisnekharka | Local |
| FARP-07 | Lakuribhanjyang | Giriraj |
| FARP-08 | Lakuribhanjyang | Giriraj |
| FARP-09 | Lakuribhanjyang | Giriraj |
| FARP-10 | Lakuribhanjyang, Guardpani | Local |
| FARP-11 | Lakuribhanjyang, Batase | Dumse |
| FARP-12 | Lakuribhanjyang, Batase | Local |
| FARP-13 | Parthali (Bethanchowk -04) | Shakini |
| FARP-14 | Parthali (Bethanchowk- 04) | Ghanti khuile |
| FARP-15 | Parthali (Bethanchowk- 04) | Shakini |
| FARP-16 | Parthali (Bethanchowk- 04) | Broiler |
| FARP-17 | Parthali (Bethanchowk- 04) | Broiler |
| FARP-18 | Parthali (Bethanchowk- 01) | Broiler |
| FARP-19 | Parthali (Bethanchowk- 01) | Local |
| FARP-20 | Parthali (Bethanchowk- 02) | Local |
| FARP-21 | Parthali (Bethanchowk- 02) | Local |
| FARP-22 | Parthali (Bethanchowk- 02) | Local |
| FARP-23 | Chalnakhel, Bosandanda (Dhakshinkali-01) | Shakini |
| FARP-24 | Chalnakhel, Bosandanda (Dhakshinkali-01) | Kaude |
| FARP-25 | Chalnakhel, Bosandanda (Dhakshinkali-01) | Giriraj |
| FARP-26 | Chalnakhel, Bosandanda (Dhakshinkali-01) | Giriraj |
| FARP-27 | Chalnakhel, Bosandanda (Dhakshinkali-01) | Kadaknath |
| FARP-28 | Chalnakhel, Bosandanda (Dhakshinkali-01) | Giriraj |
| FARP-29 | Chalnakhel, Bosandanda (Dhakshinkali-01) | Giriraj |
| FARP-30 | Chalnakhel, Bosandanda (Dhakshinkali-01) | Giriraj |
| FARP-31 | Chalnakhel, Bosandanda (Dhakshinkali-01) | Local |
| FARP-32 | Chalnakhel, Bosandanda (Dhakshinkali-01) | Kalij black |
| FARP-33 | Chalnakhel, Bosandanda (Dhakshinkali-01) | Kalij Red |
| FARP-34 | Jhor, Dhakalchaur | Local |
| FARP-35 | Jhor, Dhakalchaur | Local |
| FARP-36 | Jhor, Dhakalchaur | Local |
| FARP-37 | Jhor, Dhakalchaur | Local |
| FARP-38 | Jhor, Dhakalchaur | Broiler |
| FARP-39 | Jhor, Dhakalchaur | Local |
| FARP-40 | Jhor, Dhakalchaur | Local |
| FARP-41 | Jhor, Dhakalchaur | Local |
