# Supplementary material for: Identification and characterization of probiotics isolated from indigenous chicken (Gallus domesticus) of Nepal
Source: PLoS One. 2023 Jan 19;18(1):e0280412. doi: 10.1371/journal.pone.0280412 (PMC9851537; doi:10.1371/journal.pone.0280412)
Supplement: S2 Table — (DOCX) [file pone.0280412.s002.docx]

**S2 Table.** Laboratory results of 52 LAB isolates on antibacterial inhibition, biochemical test, and *in vitro* test for potential probiotics strains

| **Isolate ID** | **Bacterial inhibition zone sizes (mm)** | | | | | | **Biochemical tests results** | | | | ***In vitro* probiotics tests results** | | | | |
| --- | --- | --- | --- | --- | --- | --- | --- | --- | --- | --- | --- | --- | --- | --- | --- |
|  | ***Sal* spp.** | ***E. coli*** | ***S. sonnei*** | ***K. pneu*** | ***C. freundii*** | ***S. aureus*** | **Sulfur test** | **Indole test** | **Catalse test** | **Oxidase test** | **% yield of lactic acid** | **Acid tolerance at pH 3.0** | **Tolerance to 0.3% bile** | **Tolerance to 0.5 % bile** | **Tolerance to 1.0 % bile** |
| 1(1) | 9 | 8 | - | - | 9 | - | - | - | - | - | 56.3 | 8.4 | 4.8 | 10.0 | 109.0 |
| 1(2) | 9 | - | - | - | - | - | - | - | - | - | 49.5 | 10.3 | 38.8 | 31.3 | 35.1 |
| 1(4) | 8 | - | - | - | - | - | - | - | - | - | <0 | <0 | NT | NT | NT |
| 14B(1) | 13 | - | 11 | 12 | 10 | - | - | - | - | - | 47.3 | <0 | NT | NT | NT |
| 14B(2) | 14 | - | 8 | - | - | - | - | - | - | - | 49.5 | <0 | NT | NT | NT |
| 15(3) | - | - | - | - | 12 | - | - | - | + | - | NT | NT | NT | NT | NT |
| 16(1) | 14 | - | 13 | - | - | - | - | - | - | - | 42.8 | 1.9 | 20.4 | 45.0 | 119.0 |
| 17(1) | - | - | - | - | 12 | - | - | - | - | - | 54.0 | <0 | NT | NT | NT |
| 17(3) | 11 | - | - | - | - | - | - | - | - | - | 49.5 | <0 | NT | NT | NT |
| 18(1) | 15 | - | 15 | - | 13 | - | - | - | - | - | 54.0 | 2.9 | <0 | <0 | <0 |
| 2(2) | 11 | 8 | 11 | 9 | 9 | - | - | - | - | - | 74.3 | 10.2 | 61.4 | 40.7 | 151.9 |
| 2(3) | 13 | - | - | - | - | - | - | - | + | - | NT | NT | NT | NT | NT |
| 20(2) | 10 | - | - | - | - | - | - | - | - | - | 42.8 | 2.4 | 18.5 | 12.4 | 100.0 |
| 22(1) | 12 | - | 15 | - | - | - | - | - | - | - | <0 | <0 | NT | NT | NT |
| 22(2) | 11 | - | 11 | - | 8 | - | - | - | - | - | 63.0 | <0 | NT | NT | NT |
| 22(3) | - | - | 12 | - | 12 | - | - | - | - | - | 42.8 | 3.7 | 11.5 | 13.1 | 126.3 |
| 24(1) | 13 | - | 16 | - | - | - | - | - | - | - | 60.8 | 5.7 | <0 | <0 | 22.5 |
| 24(2) | 13 | - | - | - | 13 | - | - | - | - | - | 54.0 | 12.3 | <0 | <0 | 66.2 |
| 24(3) | 12 | 9 | 8 | 15 | 13 | - | - | - | - | - | 51.8 | 0 | NT | NT | NT |
| 25(1) | - | - | 12 | - | 12 | - | - | - | - | - | 49.5 | 1.2 | <0 | <0 | 133.1 |
| 25(2) | 12 | - | 11 | 14 | 12 | - | - | - | - | - | 56.3 | <0 | NT | NT | NT |
| 25(3) | 12 | - | 12 | 11 | 12 | - | - | - | - | - | 51.8 | 6.6 | <0 | <0 | <0 |
| 25B(1) | 14 | - | 10 | - | - | - | - | - | - | - | 47.3 | 48.5 | 66.6 | 52.6 | <0 |
| 25B(2) | 16 | 13 | 12 | 9 | 11 | - | - | - | - | - | 81.0 | 43.0 | 73.8 | 99.0 | <0 |
| 26(1) | - | - | 11 | 11 | - | - | - | - | - | - | 51.8 | 10.7 | <0 | <0 | <0 |
| 26(2) | - | - | - | - | 9 | - | - | - | - | - | 49.5 | 3.7 | <0 | <0 | <0 |
| **26B** | **18** | **13** | **10** | **9** | **11** | **12** | **-** | **-** | **-** | **-** | **76.5** | **51.1** | **34.8** | **0.5** | **<0** |
| 27(1) | 16 | - | - | - | 7 | - | - | - | - | - | 47.3 | 10.4 | <0 | <0 | 7.0 |
| 28B(1) | 16 | 14 | 11 | 8 | 12 | 10 | - | - | - | - | 76.5 | 36.6 | 44.5 | 2.7 | <0 |
| **28B** | **17** | **14** | **11** | **9** | **12** | **13** | **-** | **-** | **-** | **-** | **72.0** | **56.0** | **54.9** | **49.1** | **<0** |
| 29(1) | 9 | 9 | 10 | - | - | - | - | - | + | + | NT | NT | NT | NT | NT |
| 29(2) | 9 | - | - | - | - | - | - | - | + | + | NT | NT | NT | NT | NT |
| **30B** | **16** | **13** | **10** | **9** | **12** | **16** | **-** | **-** | **-** | **-** | **72.0** | **50.5** | **62.0** | **32.8** | **<0** |
| 30B(2) | 17 | 14 | 10 | 10 | 11 | 13 | - | - | - | - | 76.5 | 46.7 | 28.5 | 30.0 | <0 |
| 36(10) | 8 | - | - | - | 8 | - | - | - | - | - | <0 | <0 | NT | NT | NT |
| 36(11) | - | - | - | - | 11 | - | - | - | - | - | <0 | <0 | NT | NT | NT |
| 36(3) | 12 | - | - | - | - | - | - | - | - | - | 40.5 | 3.2 | 11.8 | 6.9 | 107.5 |
| **C4/36(4)** | **13** | **-** | **-** | **-** | **-** | **-** | **-** | **-** | **-** | **-** | **42.8** | **1.2** | **136.0** | **103.3** | **67.3** |
| 36(7) | - | - | - | - | 12 | - | - | - | - | - | <0 | <0 | NT | NT | NT |
| 36(8) | - | - | - | - | 10 | - | - | - | - | - | <0 | <0 | NT | NT | NT |
| 36(9) | - | - | - | - | 9 | - | - | - | - | - | <0 | <0 | NT | NT | NT |
| 37(1) | - | - | - | 7 | - | - | - | - | - | - | 47.3 | <0 | NT | NT | NT |
| 37(2) | - | - | - | 8 | - | - | - | - | - | - | 38.3 | <0 | NT | NT | NT |
| 37(3) | - | - | - | 8 | - | 7 | - | - | - | - | 47.3 | 62.1 | 163.6 | 164.3 | <0 |
| 38B(1) | 14 | - | - | - | - | - | - | - | + | - | NT | NT | NT | NT | NT |
| 38B(2) | 15 | - | - | - | - | - | - | - | + | - | NT | NT | NT | NT | NT |
| 39B(1) | - | 12 | - | - | - | - | - | - | - | - | 45.0 | 27.2 | 150.4 | 215.5 | <0 |
| 4(3) | 14 | 10 | 12 | 10 | 9 | - | - | - | - | - | 40.5 | 2.2 | 9.6 | <0 | 93.5 |
| 4(4) | 12 | - | - | - | - | - | - | - | - | - | <0 | <0 | NT | NT | NT |
| 41(1) | 10 | - | - | - | - | 7 | - | - | - | - | 56.3 | <0 | NT | NT | NT |
| 8(3) | - | 7 | - | - | - | - | - | - | - | - | <0 | <0 | NT | NT | NT |
| 8(4) | - | 9 | - | - | 9 | - | - | - | - | - | 51.8 | 10.6 | <0 | <0 | 96.1 |

*Sal spp.; Salmonella spp., K. pneu; Klebsiella pneumonia;* NT; not tested, +; positive test result, -; negative test result

All above 52 isolates were Gram positive rods, and produced CaCO_3_ hydrolysis clearing zones

The bold font represents the profile of potential probiotics isolates subjected to 16SrDNA sequencing analysis
