# Supplementary material for: Identification and characterization of probiotics isolated from indigenous chicken (Gallus domesticus) of Nepal
Source: PLoS One. 2023 Jan 19;18(1):e0280412. doi: 10.1371/journal.pone.0280412 (PMC9851537; doi:10.1371/journal.pone.0280412)
Supplement: Attachment — Submitted filename: Rebuttal Letter_21Sep22_edit.docx [file pone.0280412.s003.docx]

**Point by point response to editor’s and reviewer’s comments:**

1. **Please ensure that your manuscript meets PLOS ONE's style requirements, including those for file naming.**

- The file names have been corrected.

2. **a) Please clarify the sources of funding (financial or material support) for your study. List the grants or organizations that supported your study, including funding received from your institution.**

- Partial fund for conducting the laboratory experiments were received as academic thesis support from SANN International College, Purbanchal University (by the authors Mohan Gupta, Ujwal Shrestha, Saubhagya Dangol, and Sudarshan GC). Rest of the fund for conducting this study was obtained from institutions BIOVAC Nepal and Center for Molecular Dynamics Nepal (CMDN).

**b) State what role the funders took in the study.**

- The funders had role in data analysis and preparation of the manuscript study design.

**c) If any authors received a salary from any of your funders, please state which authors and which funders.**

- Roji Raut, Sulochana Manandhar, Ashok Chaudhary, Pragun Rajbhandari, and Prajwol Manandhar received salary from Center for Molecular Dynamics Nepal (CMDN).
- Rajindra Napit, and Dibesh Karmacharya received salary from BIOVAC Nepal.

3. **Question on Figure 1:**

- Figure 1 is not a copy right material. Instead, this has been created by the authors using the program Q-GIS (version 3.22.7) based on the recorded GPS locations. We permit the figure to be used freely by anyone for download, copy, distribute and any other way.

**Response to the comments of Reviewer 1**

**1. At line 4, please make sure your short title is “Isolation and identification of probiotics from native chicken breeds of Nepal” or “Isolation and identification of probiotics from chicken of Nepal”? Please keep consistent.**

- This has been corrected in the revised manuscript now by replacing with the former phrase.

**2. Please give references for sentences at line 76-77 and 77-78.**

- The reference to the first sentence was provided. For the second sentence, the use, and particularly production of probiotics in poultry and other livestock sectors in Nepal is relatively much less practiced. This sentence has been accordingly modified in the revised manuscript now.

**3. At line 107, 110, 120, 124, 190, 205, 206, 207, there should be spaces between words “with0.5%”, “India) with 0.5%”, “O.D630adjustedbacterial”, “0.5O.D630”, “fromvarious”, “37produced”, “37isolates, 27isolates”, “17isolates”. I will not list all of those kind of error, please correct throughout the manuscript.**

- We apologies for the typo. It seems like when the Microsoft word document is shared between authors who have different versions of operating system, the problem of word spacing automatically appears. We have checked all the text word by word in the revised manuscript now. Thank you for pointing this out.

**4. At line 88, there shouldn’t be a space before “Sampling”.**

- This has been corrected in the revised manuscript now.

**5. At lines 126-128, what’s the criterion you used to determine whether the LAB isolates have anti-bacterial activity or not? Although you mentioned in the results part at line 195 that “pathogen with zone of inhibition ranging from 7 to 16mm”, please give a reference, and is the same criterion (zone range) applied for every pathogen?**

- The production of clear zone of any size around the inoculated wells was taken as an indication of antibacterial activity imposed by that LAB isolate against the given pathogen inoculated as a lawn culture. This criterion has been applied for every tested pathogen. In this study, the observed zones of inhibition ranged between 7 mm to 16 mm. This has been clarified in the revised manuscript, and referenced too.

**6. How these 90 potential LAB isolates distributed among the 41 cloacal samples? How do you distinguish the LAB isolates from same cloacal/plate sample?**

- Multiple morphologically distinct colonies producing clear hydrolyzing zones and ranging from white to cream in colour and measuring 3-4 mm in size were selected as these could be potentially distinct LAB isolates. Because we used cloacal swab as source sample, it is expected to harbor diverse culturable microbiome, many of which could be potential LAB strains. This has been clarified in the revised manuscript now.

**7. At lines 199-200, please delete “of which 5 isolates also inhibited S. aureus.”, it’s repetitive.**

- This has been responded accordingly in the revised manuscript now.

**8. According to your description from lines 136-146, please make clear at line 205, the 38%-81% is acid yield or percentage of acid yield.**
- This has been clarified in the revised manuscript now.

**9. At lines 151-153, to calculate the acid tolerance, why not use two pH for same medium, here you use gastric juice media and MRS.**

- We wanted to test if the bacterial growth *invitro* in its favorable MRS medium was comparable to the actual gastric juice present *invivo*. The use of two pH for same medium would have been easier by simply adjusting the pH to 3 and 7, however with our long term motive to establish it as a feed supplement, we used gastric juice media for more realistic comparision.

**10. From line 202-209, looks like not all the LAB isolates applied to all the screening methods, please clarify how many isolates were tested in each method. In an other words, are these tests applied sequentially.**

- Yes, not all LAB isolates were subjected to all screening methods. Only those isolates testing positive in each screening step were tested further in the next following test. The order of the tests was as mentioned sequentially in the Methods section. That is, the exact sequence were the selective isolation in CaCO3 media (N=90), followed by antimicrobial susceptibility test (N=52), phenotypic test (Gram staining and biochemical tests)(N=46), and test for lactic acid production (N=37). Finally, these 37 isolates fulfilling all above screening parameters were subjected to tolerance tests to acid and bile salt.

The sequence of screening has been mentioned in methods. For further clarification, explanations have been added in the methods section under ‘Test for acid tolerance’ and ‘Test for bile tolerance’.

**11. At line 223, please change “a” to “as”.**

-This has been responded accordingly in the revised manuscript now.

**12. According to the formula at line 167, how you get value above 100% for the bile tolerance data at Table S2? Also, is that reasonable that the tolerance increases as the bile concentration increase, for example, data for 22(3)?**

- The formula for bile tolerance is based on absorbance defined by CFU. The value above 100% signifies, the bacterial number with bile has doubled faster than without bile. The increase in bile concentration doesn’t necessarily increase the bile tolerance, infact, less than 50% of the isolates showed no tolerance to bile with even lesser isolates able to tolerate 1% bile. A similar pattern was shown in the works by Anukam & Koyama, 2007 whereby the CFU increased upto 0.5% bile concentration and decreased on further increased bile cocncentration. Here, we selected the samples with highest tolerance at 0.3% and reduced bile tolerance at 0.5% and 1% bile concentration. The reason we did not select 22(3).

**13. Conclusion for C4/36(4), although it has high tolerance to bile, but one of the important characteristics of probiotics is the ability to inhibit pathogen growth, looks it doesn’t fit.**

- Although C4/36(4) doesn’t fit the most essential characteristic of pathogen growth, we have selected it for molecular screening. The isolate doesn’t seem to have any significant feature to be an isolate of choice for molecular screening as it showed no pathogen inhibition *invitro*. Still we have chosen it because, we are unaware of the behavior of the bacteria *invivo* , the actual mechanism by which the bacteria inhibits the pathogen. So, if the bacteria has a high enough tendency to tolerate bile, it may show a different behavior *invivo* with pathogen interaction; which is again still our hypothesis. So, having 3 isolates with all the desired characteristics, we have selected one entirely different isolate. The molecular screening indeed showed the isolate to be homologous to L.reuteri, one very potential probiotic candidate.

**14. It’s not necessary to put sentences at lines 301-302 in conclusion part.**

-This has been responded accordingly in the revised manuscript now.
