# Supplementary material for: Identification and characterization of probiotics isolated from indigenous chicken (Gallus domesticus) of Nepal
Source: PLoS One. 2023 Jan 19;18(1):e0280412. doi: 10.1371/journal.pone.0280412 (PMC9851537; doi:10.1371/journal.pone.0280412)
Supplement: Attachment — Submitted filename: Rebuttal_21Dec22.docx [file pone.0280412.s004.docx]

15^th^ November 2022

**Response to reviewer’s comments**

Reviewers:

1. In this revised manuscript, two main questions should be clarified. Firstly, please clarify the fundings, institutions, authors, and their roles in this paper.

**Response:** Funding: Partial fund for conducting the laboratory experiments were received as academic thesis support from SANN International College, Purbanchal University (by the authors Mohan Gupta, Ujwal Shrestha, Saubhagya Dangol, and Sudarshan GC). Rest of the logistical support for conducting this study was obtained from institutions BIOVAC Nepal and Center for Molecular Dynamics Nepal (CMDN).

The authors, their affiliated institutions, and contribution in this study are already clearly mentioned in the PLOSone manuscript submission portal during paper submission.

2. Secondly, please make a clear explanation of the equations you used in this study, it’s better to give any references if someone used that before. I checked the references you cited, they only had word description, without results/equation explanation.

**Response:**

The equations used in the study have been explained in the manuscript and cited accordingly.

3. Multiple institutions were listed, but only three of them provided fundings. Please list the roles and responsibilities of institutions from France, USA, Spain, Australia played in this paper. Also, please list the contribution of each author.

**Response:** All the other institutions provided intellectual and technical support in study design and manuscript preparation.

2. At “Financial Disclosure” region, you claimed that “The funders had no role in study design, data collection and analysis, decision to publish, or preparation of the manuscript.” But when you answered the question 1b) of the editor, you claimed that “The funders had role in data analysis and preparation of the manuscript study design”. Which is the correct one?

**Response:** The statement “The funders had role in data analysis and preparation of the manuscript and study design” is correct and amended accordingly in the paper.

3. The short title is essentially same as the title, please change it to a concise one.

**Response:** The short title has been replaced as “Characterization of probiotics from indigenous chicken”

4. At lines 78-80, the sentences should be rephrased as “However, the use and particular production of probiotics in poultry sectors in developing countries, like Nepal, is relatively less pronounced.”

**Response:** This has been corrected accordingly.

5. At lines 140, the title was “Test for production of total lactic acid”, but the formula and results you demonstrated were percentage yield of lactic acid. Please make an explanation and provide the method you used to calculate the actual yield of lactic acid.

**Response:**

Actual yield of lactic acid was calculated using the formula,

**Actual lactic acid present in 100 ml of bacterial broth = percent yield of lactic acid = (Vg × N × 90 × 100)/Vm,**

Where, Vg= volume of NaOH solution added for titration

N= concentration of sodium hydroxide standardized solution expressed in Eq/L

90= equivalent weight of lactic acid

Vm= volume of LAB broth culture used for titration

As gram equivalent weight of NaOH is 40, and we used 0.1 N of NaOH,

And, balanced chemical equation for reaction between NaOH and Lactic acid is as:

C_3_H_6_O_3_ + NaOH → NaC_3_H_5_O_3_ + H_2_O

ie. 1 mole of NaOH requires 1 mole of Lactic acid for neutralization.

Since, 1 mole of NaOH = 40gm and 1 mole of Lactic acid = 90.08g

40gm NaOH requires 90.08g Lactic acid

Ie. 1 gm of NaOH requires **2.25 gm** Lactic acid

Thus, actual lactic acid yield = (Vg X 4 X 2.25)/1000 x Vm

Here, Vm= 2 as we used 2 ml of LAB culture for initial titration.

Thus, **actual lactic acid yield** = (Vg X 4 X 2.25)/1000 x 2

= **Vg X 0.0045**

For example, for our sample 1 (1), volume of NaOH required for titration was **2.5 ml**.

Thus, % lactic acid yield for this sample 1 (1) = actual yield/theoretical yield X 100

% lactic acid yield for sample 1 (1) = (2.5 X 0.0045) X 100 = 56.25

0.02

This formula has been detailed in the article “Fabro M, Milanesio H, Robert L, Speranza J, Murphy M, Rodríguez G, et al. Determination of acidity in whole raw milk: comparison of results obtained by two different analytical methods. Journal of dairy science. 2006;89(3):859-61.” which we have cited in the paper.

6. At lines 158-160, 168-170, different bacteria have different behavior, you need to make your own curve of the relationship of CFU/ml and OD value. And the reference you cited, looks like the calculation they did was wrong, according to their equation, OD1 was equal to 7.968*10^8 CFU/ml.

**Response:**

As per the suggestion, we did perform the growth curve for one of our bacterial isolate 28B (1) that is *Lactobacillus plantarum*, and obtained the growth curve as shown in Figure 1.

Growth curve of isolate 28B(1)

OD

| Time (hr) | OD(I)-OD(B) |
| --- | --- |
| 0 | 0.033 |
| 1 | 0.069 |
| 2 | 0.082 |
| 3 | 0.082 |
| 4 | 0.095 |
| 5 | 0.082 |
| 6 | 0.076 |
| 7 | 0.091 |
| 8 | 0.082 |
| 9 | 0.104 |
| 10 | 0.088 |
| 11 | 0.086 |
| 12 | 0.094 |
| 15 | 0.099 |
| 18 | 0.102 |
| 21 | 0.106 |
| 24 | 0.127 |
| 27 | 0.146 |
| 30 | 0.301 |
| 33 | 0.449 |
| 36 | 0.583 |
| 39 | 0.567 |
| 42 | 0.57 |
| 45 | 0.575 |
| 48 | 0.548 |

Figure 1. Growth curve analysis of isolate 28B(1)

Further, we established the OD and CFU relationship as shown in Figure 2. We obtained the equation for relationship as y=7.3249+4.3782.

Log (CFU)

| OD | Log Cfu |
| --- | --- |
| 0.7665 | 9.11394 |
| 0.548 | 8.81291 |
| 0.418 | 8.51188 |
| 0.334 | 8.21085 |
| 0.2995 | 7.90982 |
| 0.2845 | 7.60879 |
| 0.2785 | 7.30776 |
| 0.273 | 7.00673 |
| 0.2705 | 6.7057 |
| 0.269 | 0 |

Figure 2. Correlation between CFU and OD

Using this equation, we could obtain similar percentage increase in bile (table 1) and acid tolerance (table 2) as we obtained using the equation from the paper (Trabelsi et.al. 2013).

Table 1. Comparison of bile tolerance using two equations

Table 2. Comparison of acid tolerance using two equations

Since no larger variation was observed in our final data, the OD and CFU correlation seems to be consistent irrespective of the reading.

7. At line 162, there should be a space between “mlat”. And the equation should be Percent acid tolerance=(CFU/ml at pH3)/(CFU/ml at pH7), if you want to mean the percent of bacteria survive in acids.

Same problem for the equation at line 171. If my understanding of these calculations is correct, you should adjust your results’ data.

**Response:**

We calculated percent acid tolerance as $\frac{CFU/ml at pH 7.0-CFU/ml at pH 3.0}{CFU/ml at pH 7.0}x100\%$ as calculated in a cited paper “Farid W, Masud T, Sohail A, Naqvi S, Qazalbash MA. Molecular characterization and 16S rRNA sequence analysis of probiotic lactobacillus acidophilus isolated from indigenous Dahi (Yoghurt). International Journal of Bioscience. 2016;9(5):19-27”

8. What’s the reason not identify LAB first (Phenotypic bacterial identification), then do the analysis of antibacterial activity?

**Response**: We thank the reviewer for the suggestion, which is in fact a standard approach. We, on the other hand, adopted a reverse approach where we first assessed the antibacterial activity followed by phenotypic bacterial identification. It was because we wanted to isolate potential LAB having antibacterial activity. In earlier pilot study, we found that large proportion of phenotypically identified LAB isolates lacked antibacterial activity. Thus, to reduce our work load and resources, we first performed antibacterial assessment of potential LAB isolates, and only those producing inhibitory activity were subjected to phenotypic bacterial identification.

9. At line 199, from Table S2, looks zone of inhibition ranging was from 7 to 18 mm.

**Response**: This has been corrected now.

10. At lines 199-201, of the six pathogenic bacteria you tested, only Staphylococcus aureus is G positive, you can describe the results you had, but it’s unrepresentative.

**Response**: Yes, we agree on this. This sentence has been removed now.

11. At line 214, where did the number “31” come from? Since only 27 isolates from the 37 acid-producing isolates can survive in pH3.

**Response**: This has been corrected now.

12. Table S2, table note, please change CaCO3 to CaCO3.

**Response**: This has been corrected now.
